# Anti-melanoma effect and action mechanism of a novel chitosan-based composite hydrogel containing hydroxyapatite nanoparticles

**DOI:** 10.1093/rb/rbac050

**Published:** 2022-07-29

**Authors:** Kejia Xu, Yifu Wang, Yao Xie, Xiaoyan Zhang, Wei Chen, Zhongtao Li, Tingting Wang, Xiao Yang, Bo Guo, Lin Wang, Xiangdong Zhu, Xingdong Zhang

**Affiliations:** Department of Dermatovenereology, West China Hospital, Sichuan University, Chengdu 610041, China; National Engineering Research Center for Biomaterials, Sichuan University, Chengdu 610041, China; Department of Dermatovenereology, West China Hospital, Sichuan University, Chengdu 610041, China; Department of Dermatovenereology, West China Hospital, Sichuan University, Chengdu 610041, China; Department of Dermatovenereology, West China Hospital, Sichuan University, Chengdu 610041, China; Department of Dermatovenereology, West China Hospital, Sichuan University, Chengdu 610041, China; Department of Dermatovenereology, West China Hospital, Sichuan University, Chengdu 610041, China; National Engineering Research Center for Biomaterials, Sichuan University, Chengdu 610041, China; Department of Ophthalmology, West China Hospital, Sichuan University, Chengdu 610041, China; Department of Dermatovenereology, West China Hospital, Sichuan University, Chengdu 610041, China; National Engineering Research Center for Biomaterials, Sichuan University, Chengdu 610041, China; National Engineering Research Center for Biomaterials, Sichuan University, Chengdu 610041, China

**Keywords:** melanoma, hydroxyapatite nanoparticles, composite hydrogel, tumor inhibition, biosafety

## Abstract

Hydroxyapatite nanoparticles (HANPs) have been increasingly regarded and reported due to their potential anti-tumor ability. Previously, we found that the rod-like HANPs had good application potential for cutaneous melanoma (CMM). To satisfy the actual requirements in repairing post-operative skin defects and inhibiting CMM recurrence after tumorectomy, we constructed a novel chitosan/alginate (CS/Alg) hydrogel containing the aforementioned HANPs. The *in vitro* cell experiments confirmed that activated mitochondrial-dependent apoptosis was tightly related to the anti-tumor ability of HANPs. Specifically, we further discovered several target proteins might be involved in abnormal activating Wnt, proteoglycans in cancer, oxidative phosphorylation and p53 signaling pathways. The *in vivo* animal experiments demonstrated that the HANPs-loaded CS/Alg hydrogel (CS/Alg/HANPs) had a similar effect on inhibiting tumor growth as HANPs, and CS/Alg hydrogel as well as phosphate buffered saline (PBS) group (control) not showed any effect, proving the key role of HANPs. The immunohistochemical staining demonstrated a tumor inhibition via the mitochondria-mediated apoptosis pathway, consistent with the *in vitro* evaluation. Moreover, CS/Alg/HANPs exhibited no additional biosafety risk to the functions of major organs. Overall, this CS/Alg/HANPs hydrogel has substantial application potential for treating CMM.

## Graphical Abstract

**Figure:**
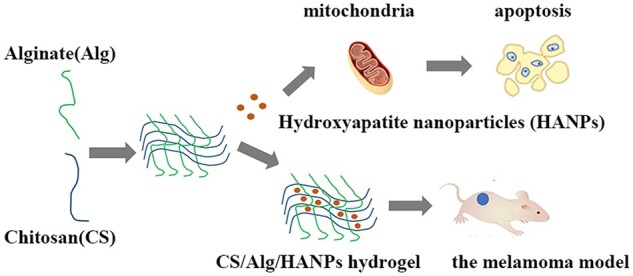

## Introduction

Cutaneous melanoma (CMM), characterized by high metastatic potential, is the most lethal type of skin tumor, and its annual incidence is continuously increasing in most populations worldwide [1].

One estimate suggests that ∼324 635 new skin melanoma cases and 57 043 new melanoma-related deaths occurred in 2020 [2].

Conventional clinical treatment strategies for CMM include definitive surgical resection and adjuvant options such as radiotherapy, chemotherapy, targeted therapy and immunotherapy to reduce recurrence and thus improve patient survival rates post-surgery. Tumor relapse after surgery is a substantial risk for patients with local disease (early-stage IIB and Stage IIB), node-positive disease (Stage III), and resectable advanced/metastatic disease (Stage IV) according to TNM staging [3, 4]. However, adjuvant approaches present problems, including lack of tumor sensitivity, damage to normal tissues, poor tolerance and high costs [5–7]. In addition, skin cancer-impaired wound healing, frequent post-surgical bacterial infection and skin regeneration complications can arise during and after surgical resection, resulting in pain, extended hospital stays and increased expenses [8]. Due to the above considerations, the development of multifunctional biomaterials that can be combined with anti-tumor and skin tissue regeneration treatments after CMM resection is urgently needed.

An increasing number of recent studies have indicated that hydroxyapatite nanoparticles (HANPs), natural nanoscale calcium apatite compounds that play an important role in hard tissue engineering and act as drug carriers [9], also exhibit notable specific anti-cancer activities [10]. It has been reported that HANPs could efficiently kill several types of tumor cells, including hepatoma cells (Bel-7402, HepG2) [11, 12], gastric cancer cells (MGC-803, SGC-7901) [11–13], breast cancer cells (4T1, MDA-MB-23, MCF-7) [14–16], lung cancer cells (A549) [17], osteosarcoma cells (Os-732) [12, 18], glioma cells (U251, SHG44) [19] and colon cancer cells (HCT116) [20]; additionally, they presented a negligible impact on certain normal cells, such as hepatic cells (L-02) [12] and lung fibroblasts (MRC-5) [17]. HANPs entered cells through the caveolin-mediated pathway or the clathrin-dependent endocytosis process via clathrin-associated protein adaptin-2 (AP2), further stimulating apoptosis and suppressing tumor proliferation, migration or invasion [21]. The underlying molecular mechanisms remain unclear, but several factors have been presumed to be involved. First, the tumor inhibition ability of HANPs highly depends on their physicochemical properties, including shape and size [12]. Second, HANPs exhibited distinct anti-tumor therapeutic effects in different types of cancer cells. The cytoplasmic internalization of nanoparticles (NPs) by highly malignant cells is greater than that by cells with low malignancy [22]. In addition, several changes in the local microenvironments, such as calcium overload, oxidative stress and activation of the mitochondrion-mediated apoptosis pathway were commonly observed in some types of tumor cells [15, 23].

Li *et al*. [24] first reported that HANPs inhibited the proliferation of melanoma cells in 2008. Later, Guo *et al*. [25] observed a similar phenomenon *in vitro* and further proposed that this anti-melanoma effect was mainly influenced by the size of HANPs. Subsequently, Wu *et al*. [26] found that granular HANPs significantly promoted apoptosis in A375 melanoma cells. Our research group has indicated that rod-like HANPs had the most prominent suppressive effect. They suppressed melanoma tumor proliferation both *in vitro* and *in vivo* and enhanced the viability of normal skin fibroblasts, indicating a potential for their use in the post-operative treatment of CMM [27]. However, whether the HANPs induce apoptosis and corresponding microenvironment alterations, along with the possible underlying mechanisms, require further investigation. Furthermore, the aggregation of HANPs is a major challenge in clinical applications [26]. In addition, there is no definitive evidence that HANPs accelerate skin wound healing; thus, HANPs may not meet post-operative patient needs. Hydrogels have long been considered promising materials for wound healing due to their porous structure [28]. Chitosan (CS) is derived from the deacetylation of chitin and alginate (Alg) is mainly extracted from brown seaweed or bacterial sources, both of which are commonly used to prepare hydrogels for wound healing [29, 30]. Nevertheless, hydrogels are difficult to handle because of their low mechanical strength and strong burst release within the first 2 days [31, 32]. Nanocomposite hydrogels (NHs) are composite materials formed by dispersing NPs in hydrogels, yielding unique physicochemical and biological properties that neither of the two building blocks can achieve independently; thus, NHs have broad application prospects in biomedicine and cancer therapy, especially for localized application [33].

Thus, in this study, we apply a previously unreported strategy for treating CMM by endowing a novel nanocomposite hydrogel with the aforementioned HANPs incorporated into the matrix, including CS and Alg, leading to an anti-tumor function. We first demonstrated that the synthesized HANPs-loaded CS/Alg hydrogel could significantly suppress melanoma development *in vivo*, while no effect was observed when the polymer structure of the CS/Alg hydrogel was used alone, proving the crucial role of HANPs. Furthermore, the CS/Alg/HANPs hydrogel had good biocompatibility and biosafety. The combination of CS/Alg hydrogel and HANPs for this application is innovative and it might be beneficial for treating local CMM recurrence while also providing a hydrated and anti-bacterial environment to facilitate wound healing. This is the first report of an anti-melanoma HANPs-loaded hydrogel system that does not serve as a drug carrier to the best of the author’s knowledge. We hope this work will provide a foundation for future clinical trials of the HANPs-containing hydrogel as a safe anti-melanoma candidate for post-surgical patients.

## Materials and methods

### Preparation of the CS/Alg/HANPs hydrogel

HANPs was supplied by the National Engineering Research Center for Biomaterials, Sichuan University, China. The traditional wet chemical reaction of Ca (NO_3_)_2_·4H_2_O, (NH_4_)_2_HPO_4_ and NH_3_·H_2_O, followed by a special post-treatment for the deposits (300°C calcination and 200-mesh screening for the freeze-dried slurry) was used for the preparation of HANPs, as described in our previous study [23]. Alginate (Alg) was obtained from Qingdao Mingyuehaizao Co. Ltd and CS was purchased from Zhejiang jinke pharmaceutical Co. Ltd. Alg (1 g) was dissolved into deionized (DI) water (200 ml) under vigorous stirring (150 rpm) for 2 h. Meanwhile, CS was quickly cleaned with DI water and vacuum-dried at 50°C for 5 h. Then, 100 ml DI water was added to the dried CS (5 g), followed by addition of glycerol (10 g) with constant stirring (300 rpm) for 2 h to obtain the yellow solution. The mixture solution was then incorporated into the above Alg solution, stirring at 300 rpm for 1 h. Finally, HANPs (98.5 g) were dispersed into the CS/Alg colloidal solution with constant stirring (100 rpm) for 3 h to obtain a milky-white CS/Alg/HANPs hydrogel. The CS/Alg hydrogel was obtained without addition of HANPs. Prior to use, both hydrogels were subjected to irradiation sterilization.

The morphologies of HANPs and the freeze-dried CS/Alg/HANPs or CS/Alg hydrogel were observed by transmission electron microscopy (TEM, Tecnai G2 F20 S-TWIN, FEI) and scanning electron microscopy (SEM, Hitachi S-4800, Japan), respectively.

### Cell culture and treatment

SK-MEL-28 and A375 human malignant melanoma cell line was performed as previously described [27].  The human keratinocytes (HaCaT) cell line was supplied by Guangzhou Cellcook Biotech Co., Ltd (China). Briefly, they were cultured in 89% Dulbecco’s Modified Eagle’s Medium (Gibco, USA) supplemented with 10% fetal bovine serum (Gibco) and 1% penicillin–streptomycin (Gibco). Then, the cells were observed with a fluorescence microscope (AX10 imager A2/AX10 cam HRC, Zeiss, Germany). Before the experiments, the HANPs solution freshly prepared was ultrasonicated for 10 min by an ultrasonic cleaning machine (Xinzhi Biotech Co., Ltd, Ningbo, China). Subsequently, cells were pretreated with different concentrations (0, 60, 120, 240 and 480 μg/ml) of HANPs suspensions.

### Cell apoptosis

According to the manufacturer’s instructions, apoptosis was assessed by Hoechst 33258 (BD Bioscience, USA) and Annexin V-FITC/PI double-staining detection kit (BD Bioscience). The protein expression treated with HANPs was further analyzed with western blotting (WB) analysis. In brief, SK-MEL-28 cells, A375 cells and HaCaT cells were seeded in six-well plates at a density of 1.2 × 10^5^, 1.0 × 10^5^ and 0.8 × 10^5^ cells/well, respectively. After incubated for 1 day, the medium was discarded, followed by the addition of various HANPs suspensions containing fresh culture medium. Coculturing 2 days later, the cells were then fixed with 4% paraformaldehyde (250 µl/well), rinsed with phosphate buffered saline (PBS), stained by Hoechst 33258 (5 mg/ml) and observed by fluorescence microscope (Leica, Germany). At the same time, the cells were harvested, washed with pre-cooled PBS, and resuspended in 500 µl of binding buffer containing 5 µl Annexin V-FITC and 5 µl PI and analyzed by flow cytometry. Then, proteins were extracted with lysis buffer (Sangon), fractionated on sodium dodecyl sulfate-polyacrylamide gels and transferred to polyvinylidene fluoride membranes. Subsequently, the membranes were incubated with primary antibodies and the secondary antibody. Protein signals were detected with BeyoECL Plus by a gel-imaging system (Bio-Rad). All antibodies were listed as follows: Bax (1:2000), Bad (1:1000), Bcl-2 (1:1000), Cytochrome C (Cyt C) (1:1000) and p53 (1:1000), which were obtained from Cell Signaling Technology (USA) and βâ-actin (1:200), anti-rabbit (1:7000), which were acquired from Santa Cruz Biotechnology (Beijing, China).

### Morphology assay

After 1 day cultured with HANPs, the cells were collected, fixed with 0.5% glutaraldehyde and osmic acid overnight at 4°C. The samples were then treated according to the general TEM protocols. Subsequently, the alterations in the nucleus, organelle and cytoplasm were examined by TEM (Hitachi, Tokyo, Japan).

### Biochemical analysis of antioxidant status

The intracellular levels of glutathione (GSH), superoxide dismutase (SOD), catalase (CAT), and malondialdehyde (MDA) were measured, respectively, according to the manufacturer’s instructions (Nanjing Jian Cheng Bioengineering Institute, China).

### Tandem mass tag-based quantitative proteomics and WB analysis

SK-MEL-28 cells were seeded in 60 mm culture dishes at a density of 6 × 10^5^ cells/dish with 240 µg/ml HANPs and without HANPs. Each group contained three parallel samples. One day later, cells were ground with liquid nitrogen and peptides were processed according to the manufacturer’s protocol for the tandem mass tag (TMT) kit (Thermo Scientific, USA). The differentially expressed proteins (DEPs) were satisfied with the following conditions: unique peptides ≥1 with average ratio-fold change ≥1.5 (up-regulation) and ≤0.67 (down-regulation), as well as *P* values ≤ 0.05. Furthermore, the DEPs were analyzed by the Kyoto Encyclopedia of Genes and Genomes (KEGG) database. The pathway with a corrected *P* value < 0.05 was considered significant. As mentioned in Cell apoptosis part, insulin-like growth factor binding protein 3 (IGFBP3) (1:1000), IGFBP5 (1:1000), thrombospondin 1 (THBS1) (1:1000), apolipoprotein A1 (APOA1) (1:1000), Dickkopf 1 (DKK1) (1:1000) and Wnt5a (1:1000) which were purchased from Abcam (USA) were analyzed by WB analysis.

### Animal model

Forty healthy Balb/c nude mice (female, 4–5 weeks, weight = 17.88 ± 0.76 g) were purchased from Beijing Huafukang Biotechnology Co., Ltd, China. The Animal Ethics Committee approved all the experiments of West China Hospital, Sichuan university (Ethical approval No. 2017088A) according to the experimental protocol. Firstly, SK-MEL-28 cells (3 × 10^6^) or A375 cells (2 × 10^6^) were resuspended in 100 μl of cell suspensions with Matrigel (50 μl, BD, USA) and were subcutaneously injected into the right-back of each mouse. Until the xenograft reached approximately about 40 mm^3^, they were anesthetized by intraperitoneal 0.3% sodium pentobarbital (Jinzhong Medical Devices Co., Ltd, Shanghai, China). Then 0.046 g HANPs, 200 µl CS/Alg/HANPs hydrogel, 200 µl CS/Alg hydrogel and 200 µl PBS were subcutaneously implanted into the tumor, respectively. The implantation should be gentle, fast (about 10 min) and the temperature was kept by 37°C throughout the process. Thirty minutes later, the mice woke up and we observed the general conditions including diet, spirit and exercise. The tumor volume of the animals was measured every 3 days until the 28th day.

### Histopathological staining, immunofluorescent staining and immunohistochemical staining

The Day 28 was considered as an experimental endpoint. The tumors were weighed after mice were sacrificed. The removed tumors were used to prepare paraffin sections, which were then performed immunofluorescent (IF) staining for terminal deoxynucleotidyl transferase-mediated deoxy uridine triphosphate nick end labeling (TUNEL) (Roche, Germany) and immunohistochemical (IHC) staining of tumor apoptosis-related markers: p53 (1:400), Cyt C (1:400), Bcl-2 (1:400), which were obtained from Bioss (China) and Bax (1:100), which was purchased from Proteintech (USA). Quantification data were performed by calculating the mean optical density value of positive-staining areas versus the whole areas in five randomly selected fields using Image-Pro Plus software (Media Cybernetics).

### Biosafety assessment

General condition including body weight, activity was observed daily. Before sacrifice, 100 μl blood was collected from each mouse for hematological and biochemical analysis. The tissue samples including heart, liver, spleen, kidney and lung were harvested and observed macroscopically and microscopically. The hematological parameters, including red blood cell count (RBC), white blood cell count (WBC), platelet count (PLT), hemoglobin (HGB), eosinophils (EOS), neutrophils (NEUT) and lymphocytes (LYM) were measured by a hematological autoanalyzer (ADVIA 2120i, Siemens, Germany). The biochemical analysis of the serum samples was carried out by an automatic biochemistry analyzer (cobasc 311, Roche, Switzerland). Serum alanine aminotransferase (ALT), aspartate aminotransferase (AST), creatinine (CREA), blood calcium (Ca) and blood glucose (Glu) were measured as well.

### Statistical analyses

The statistical results were expressed as mean ± standard deviation (SD) of three independent experiments and were analyzed by GraphPad prism 8.0. All results were analyzed by one-way analysis of variance (ANOVA) to calculate the statistical significance of differences between more than two groups. *P* < 0.05 was considered statistically significant.

## Results

### Morphological observation of the materials

**Figure 1.**
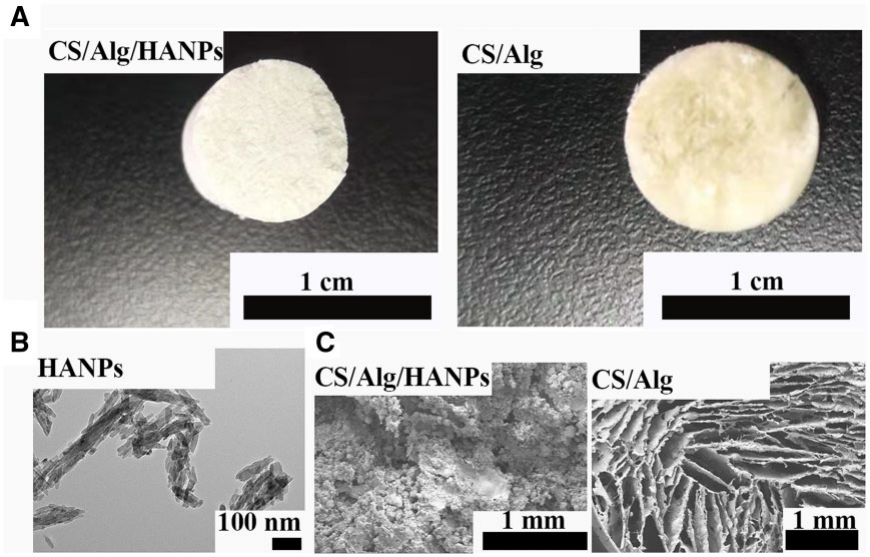
Characterization of the HANPs and the hydrogels. (**A**) The appearance of freeze-dried CS/Alg/HANPs hydrogel and CS/Alg hydrogel. (**B**) Representative TEM images of the HANPs. (**C**) SEM images of CS/Alg/HANPs hydrogel and CS/Alg hydrogel.

As shown in Fig. 1A, the CS/Alg/HANPs hydrogel is milky-white, and the CS/Alg hydrogel is yellowish-white. The HANPs were rod shaped (Fig. 1B). Both CS/Alg/HANPs and CS/Alg hydrogels had highly porous structures, indicating their potential to facilitate the effective exchange of substances, such as nutrients and oxygen (Fig. 1C).

### Cell apoptosis after coculture with HANPs and induction of mitochondria-dependent apoptosis

**Figure 2.**
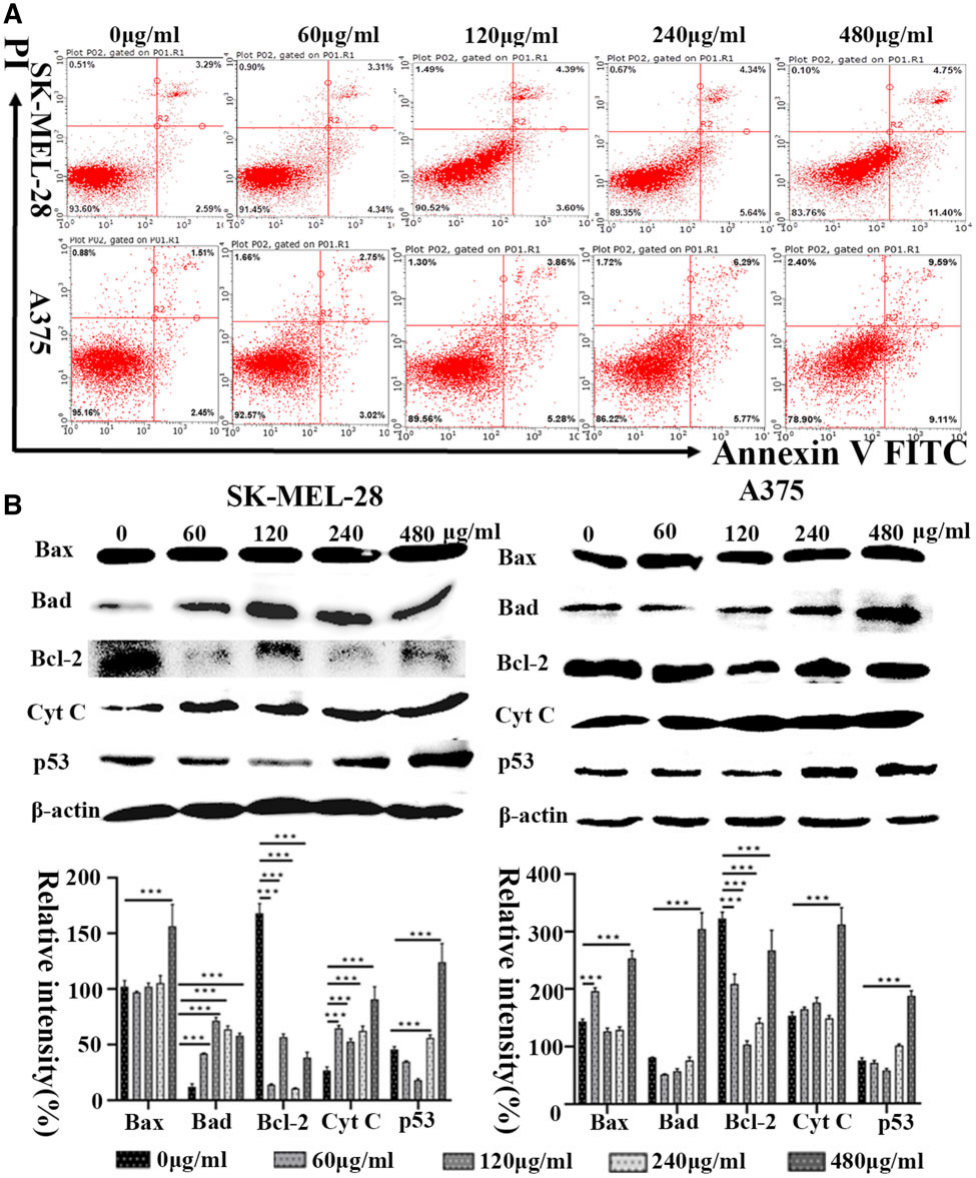
*In vitro* cell apoptosis cocultured with HANPs suspensions at various concentrations in SK-MEL-28 and A375 cells. (**A**) Density maps of annexin V-FITC/PI apoptosis detection results. (**B**) WB and quantitative analysis of the expression of apoptosis-related markers. Error bars represent SD (n = 3). ***P ≤ 0.001 compared with 0 μg/ml.

First, we detected morphological evidence of apoptosis with HANPs at various concentrations (0, 60, 120, 240 and 480 µg/ml) by Hoechst 33258. In SK-MEL-28 and A375 cells, bright blue fluorescence at different intensity levels was observed. In particular, nuclear shrinkage, condensation and fragmentation could be seen (Supplementary Fig. S1). The apoptosis rates are quantified using a flow cytometer after Annexin V FITC/PI staining (Fig. 2A). Only a small number of apoptotic cells were observed in the control groups without HANPs. However, after culturing with HANPs (240 and 480 µg/ml), the index of SK-MEL-28 cells in early and total apoptosis was notably increased. The total apoptotic indexes in A375 cells were obviously increased in a dose-dependent manner. The percentage of total apoptotic cells increased from 3.75 ± 0.26% to 18.00 ± 0.79% as concentrations increased from 0 to 480 µg/ml. The highest percentages of early and total apoptotic cells after treatment with HANPs were ∼9.11% and 18.7%, respectively, at a concentration of 480 µg/ml. Apoptosis-related proteins were subsequently investigated. Figure 2B demonstrates that the upregulation of Bax, Bad, Cyt C and p53 expression and the downregulation of Bcl-2 expression correlated with the increase in HANPs concentration in tumor cells. However, no similar phenomenon was observed in any group of HaCaT cells (Supplementary Fig. S2).

### Morphology assay by TEM

**Figure 3.**
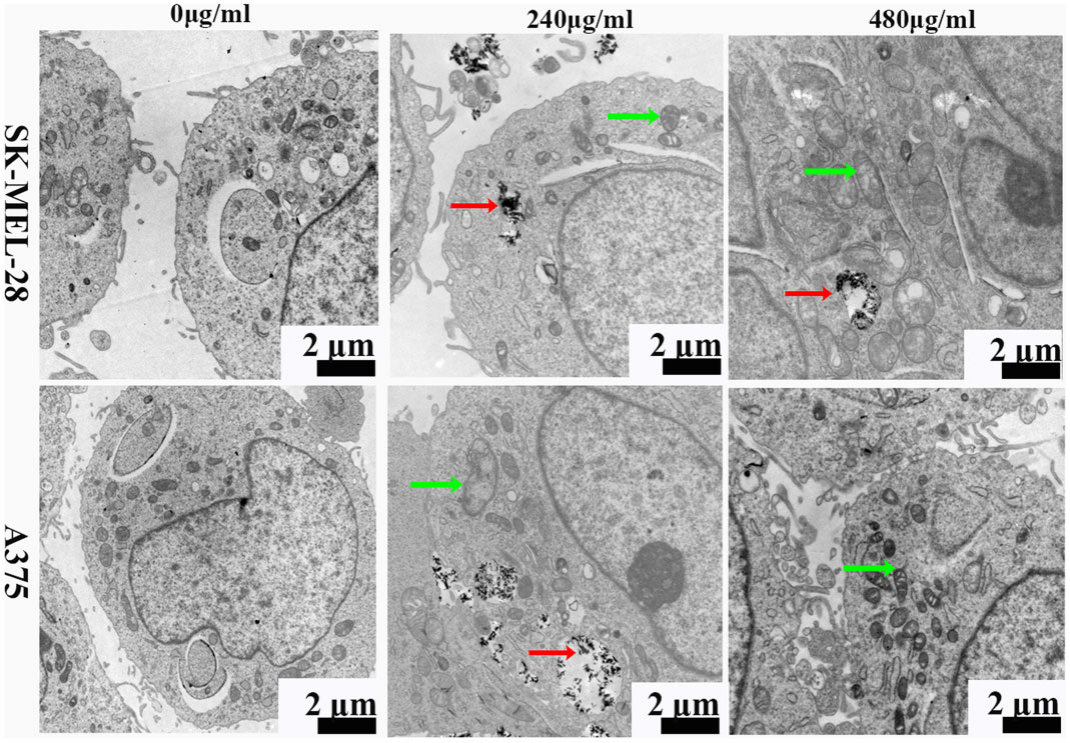
TEM micrographs of SK-MEL-28 and A375 cells treated with 0, 240 or 480 μg/ml of autoclaved HANPs. Red arrow, HANPs. Green arrow, mitochondrial swelling. Scale bars, 2 µm.

Observation of subcellular morphological alterations can contribute to a clearer understanding of the biological impact of HANPs. Mitochondrial swelling and a decrease in mitochondrial cristae were observed in tumor cells after 1 day exposure to HANPs (240 and 480 µg/ml) (Fig. 3). On the other hand, no similar ultrastructural alterations were observed in HaCaT cells (Supplementary Fig. S3).

### Oxidative stress-related biomarker assay

Intracellular redox is maintained by balancing oxidant and antioxidant substances, mainly occurs in the mitochondria. Thus, we measured reliable indicators of oxidative stress, including GSH, CAT, SOD and MDA (Supplementary Fig. S4). HANPs exposure caused an evident increase in MDA level and a decrease in GSH, CAT and SOD levels. However, these phenomena were not observed in HaCaT cells.

### TMT-based quantitative proteomic analysis

**Figure 4.**
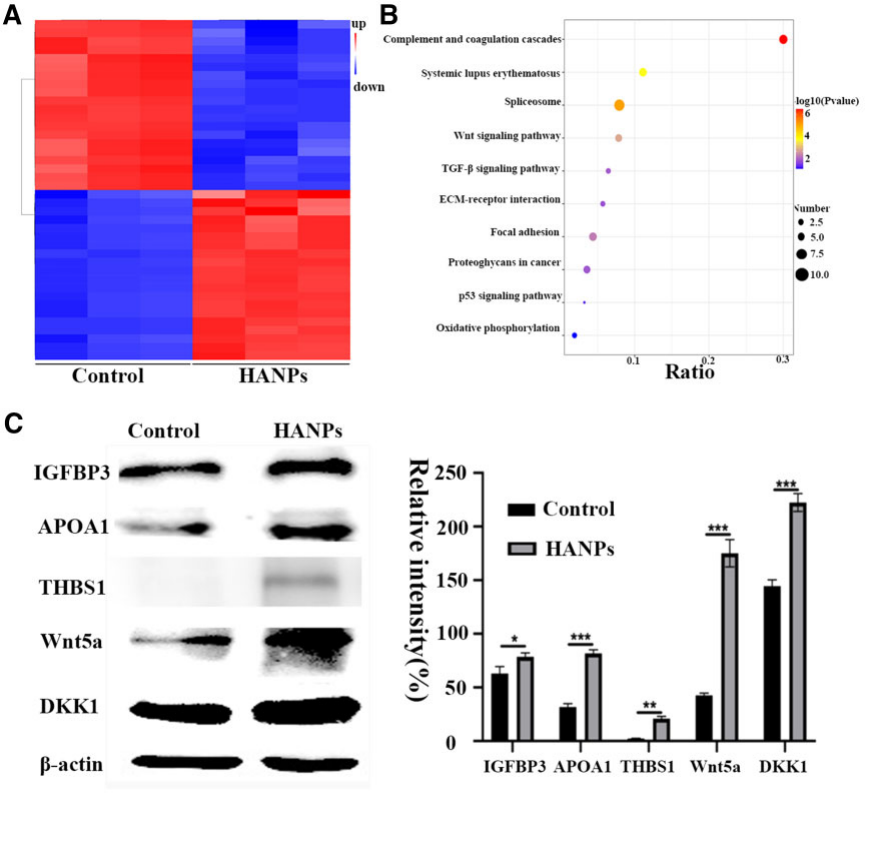
Proteome-wide changes treated with HANPs (240 μg/ml) when compared with the non-treated (0 μg/ml) control in SK-MEL-28 melanoma cells. (**A**) Heatmap clusters of the top 40 DEPs. (**B**) The top 10 altered signaling pathways based on KEGG enrichment analysis. (**C**) WB verification of the identified DEPs. Error bars represent SD (n = 3). *P < 0.05 versus control, **P < 0.01 versus control, ***P < 0.001 versus control.

We performed TMT-based quantitative proteomics sequencing and bioinformatics analysis to explore the anti-melanoma mechanisms of HANPs. A total of 7123 quantifiable proteins were identified, and 130 (62 upregulated and 68 downregulated) DEPs were identified in the treated group (240 µg/ml) versus the control group (Supplementary Fig. S5). The top 40 proteins associated with the most significant change are shown in Fig. 4A and Supplementary Table S1. The KEGG pathway analysis results are then ranked, and the top 10 enriched pathways are shown in Fig. 4B. Wnt, p53, proteoglycans in cancer and oxidative phosphorylation signaling pathways were noted. According to Refs [40, 41], we identified candidate target proteins including Wnt5a, IGFBP3, IGFBP5, APOA1, THBS1 and DKK1. The expression levels of these proteins were further verified by WB analysis to confirm the proteomics sequencing results. Consistent with the proteomic data, Wnt5a, IGFBP3, APOA1, THBS1 and DKK1 levels showed significantly higher expression after treatment with HANPs (Fig. 4C). However, the expression level of IGFBP5 showed the opposite pattern.

### *In vivo* tumor inhibitory capacity of HANPs and HANPs-loaded hydrogel

**Figure 5.**
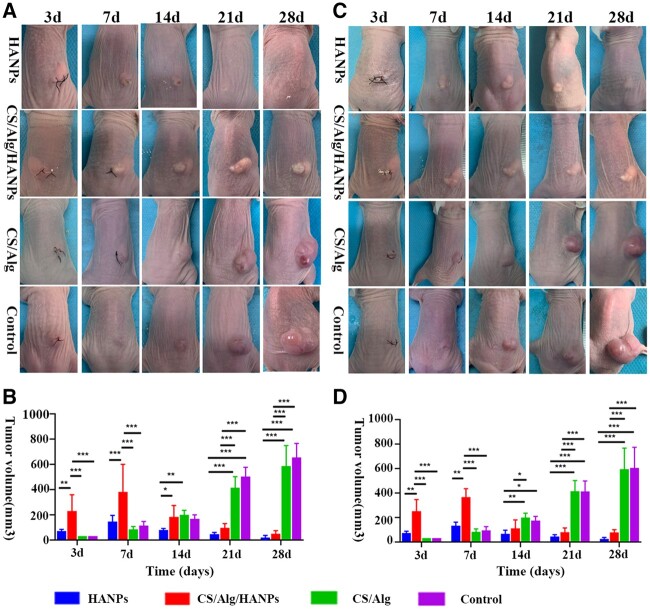
The *in vivo* tumor inhibition efficacy of HANPs and HANPs-loaded hydrogel. After implanting HANPs, CS/Alg/HANPs hydrogel, CS/Alg hydrogel and PBS (control), respectively, tumor growth (**A**) and quantification of tumor volume (**B**) on Day 3, 7, 14, 21 and 28. In A375 melanoma-bearing Balb/c nude mice, tumor growth (**C**) and quantification of tumor volume (**D**) over time similarly. Error bars represent SD (n = 5). *P < 0.05 versus control, **P < 0.01 versus control, ***P < 0.001 versus control.

**Figure 6.**
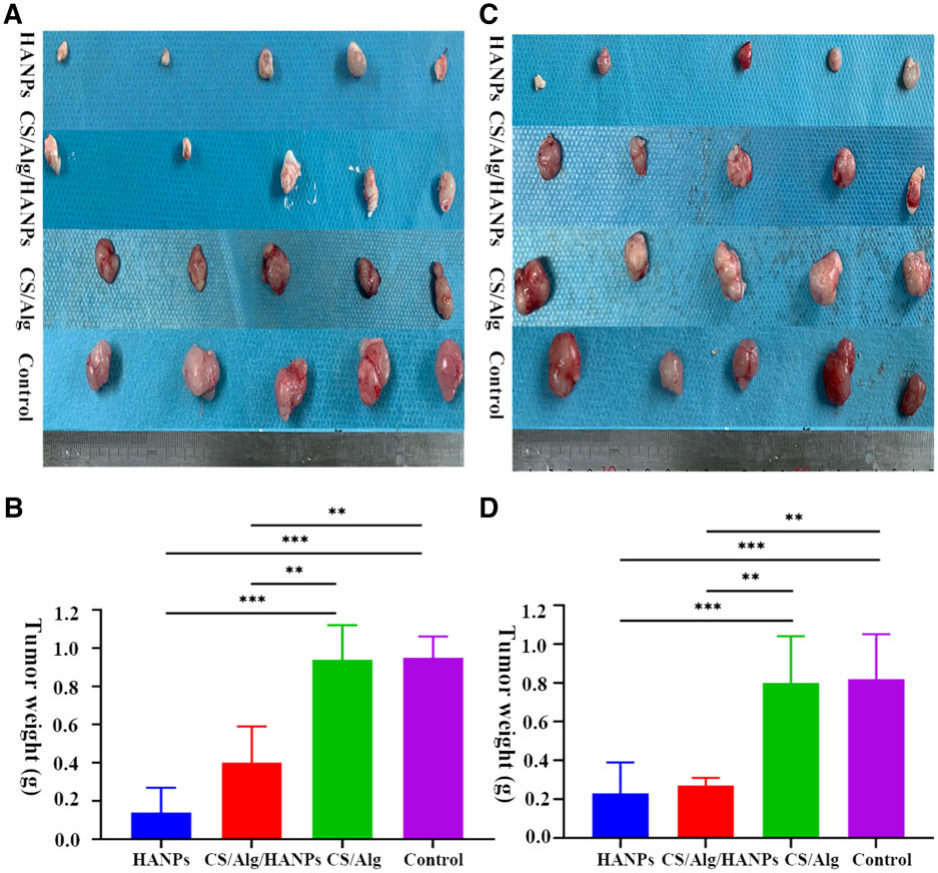
The resected tumor tissue after treated with HANPs, CS/Alg/HANPs hydrogel, CS/Alg hydrogel and PBS (control), respectively. Macroscopic observation (**A**) and weight quantification (**B**) in SK-MEL-28 melanoma-bearing Balb/c nude mice. In A375 melanoma-bearing Balb/c nude mice, observation (**C**) and weight quantification (**D**). Error bars represent SD (n = 5). *P < 0.05 versus control, **P < 0.01 versus control, ***P < 0.001 versus control.

We performed preliminary *in vivo* studies to confirm the anti-tumor effect of HANPs and HANPs-containing hydrogel to evaluate their potential clinical applications. An obviously smaller tumor volume than that in the control group and CS/Alg group was present in the HANPs and CS/Alg/HANPs groups on the 28th day in SK-MEL-28 and A375 melanoma-bearing mice. The tumors in the HANPs group were visibly smaller on the 14th day, followed by those in the CS/Alg/HANPs group on the 21st day (Fig. 5A and C). Interestingly, a large-small-large tumor volume in the CS/Alg/HANPs group was observed, which may be related to the physical properties of the hydrogel. As shown in Fig. 5B and D, there are no significant differences between the HANPs and CS/Alg/HANPs groups or the CS/Alg and PBS groups (*P* > 0.05). These results demonstrated that the CS/Alg hydrogel had no anti-melanoma ability; the anti-tumor effects of HANPs and CS/Alg/HANPs were comparable, but the onset time of the impact differed. At the endpoint, a smaller tumor volume was observed in the HANPs and CS/Alg/HANPs groups, while a larger tumor volume was found in the control and the CS/Alg groups (Fig. 6A and C). Furthermore, tumor weight also supported the results above (Fig. 6B and D). The tumors’ hematoxylin and eosin (H&E) staining is shown in Supplementary Fig. S6a. Patchy edema and fewer tumor cells were observed in the HANPs and CS/Alg/HANPs groups. Basophilic calcified deposits of HANPs and eosin-like staining speculated as the hydrogel matrix were observed.

### Activation of the mitochondrial apoptosis pathway by HANPs and HANPs-loaded hydrogel *in vivo*

**Figure 7.**
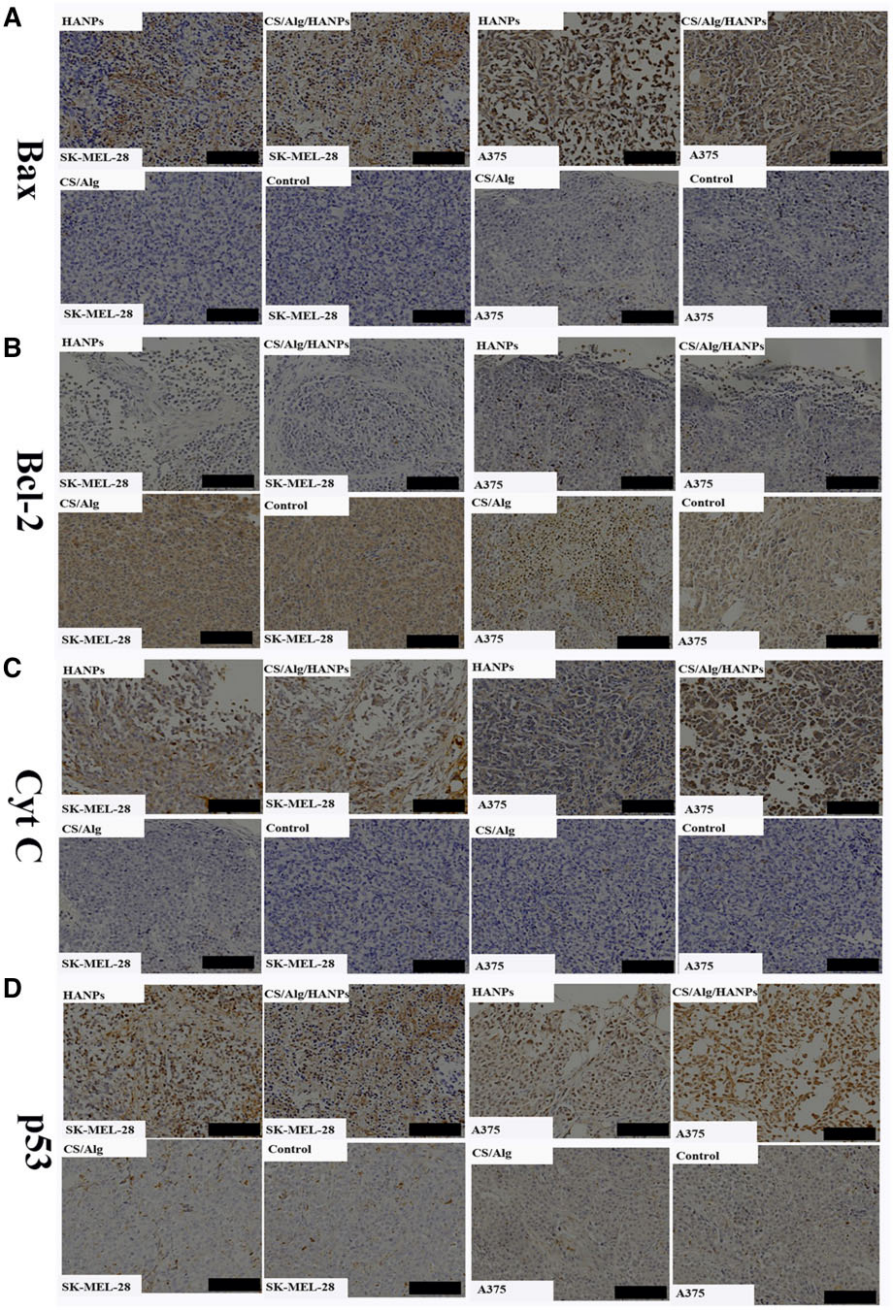
Activation of the mitochondrial apoptosis pathway by HANPs and HANPs-loaded hydrogel. The IHC analyses of Bax **(A)**, Bcl-2 (**B**), Cyt C (**C**) and p53 (**D**) of tumor tissues after different treatments. Scale bars, 100 µm, n = 5.

We performed TUNEL staining to examine whether apoptosis was involved *in vivo*. Increased TUNEL staining was observed in the HANPs and HANPs-loaded hydrogel (Supplementary Fig. S6b). Furthermore, markers associated with the mitochondrial apoptosis pathway were presented. In the Bcl-2 family, proteins Bax and Bcl-2 maintain balance in the mitochondrial apoptotic pathway. Bax is a pro-apoptotic protein and Bcl-2 is an anti-apoptotic protein; together, they regulate the release of the apoptotic factor Cyt C from the mitochondria into the cytosol [23]. DNA damage can elevate p53 level, ultimately inducing cell apoptosis to some extent [26]. Our results demonstrated that HANPs and CS/Alg/HANPs induced significantly higher levels of Bax, Cyt C, p53 and lower levels of Bcl-2 protein, suggesting that treatment with HANPs or composite HANPs hydrogel can induce intrinsic apoptosis *in vivo* (Fig. 7A–D; Supplementary Fig. S7).

### Biosafety analysis of HANPs and the HANPs-loaded hydrogel *in vivo*

**Figure 8.**
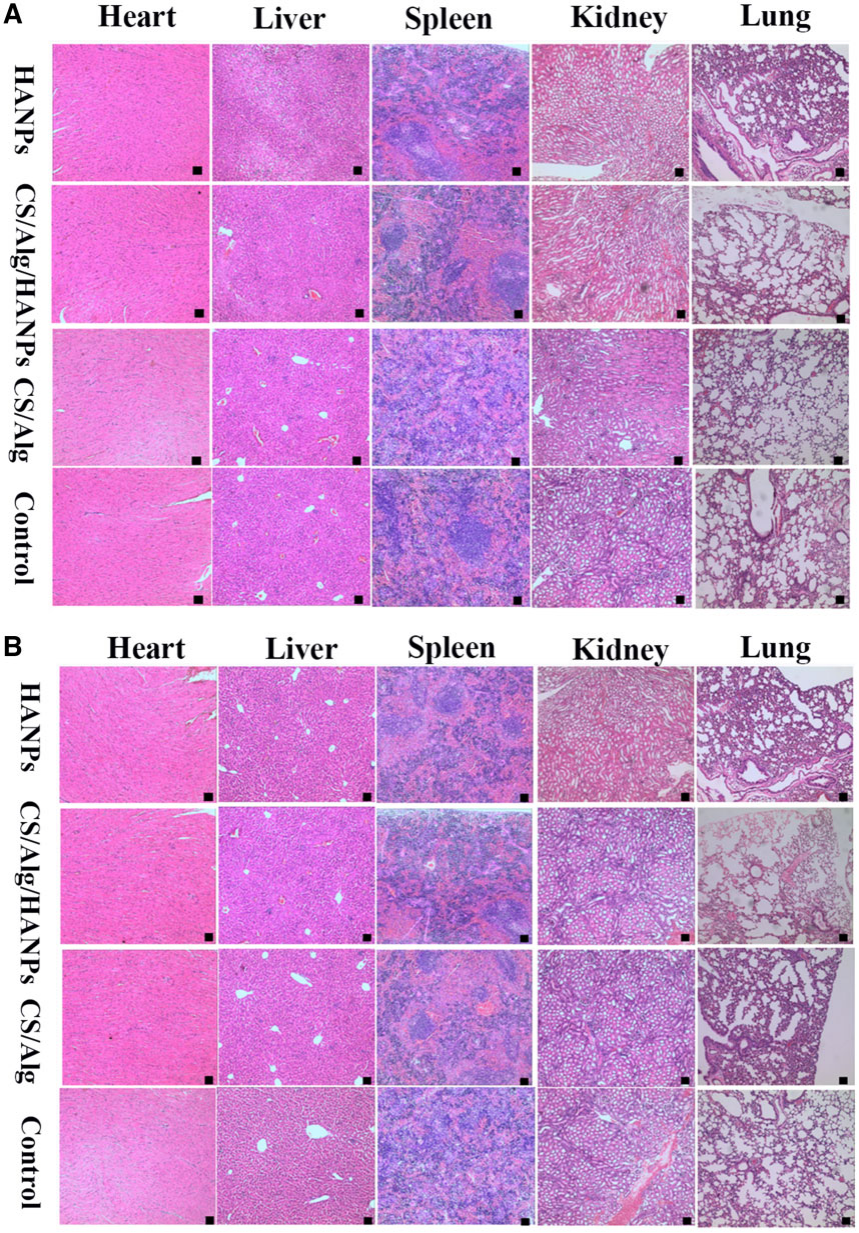
H&E staining analysis of different organs administered with HANPs, CS/Alg/HANPs hydrogel, CS/Alg hydrogel and PBS (control), respectively. (**A**) SK-MEL-28 melanoma-bearing mice and (**B**) A375 melanoma-bearing mice. Scale bars, 20 µm, n = 5.

No visible signs of toxicity (e.g. appetite loss and fatigue) were observed in any animals during treatment. Slight weight loss was observed after implantation, but the weights rapidly rebounded (Supplementary Fig. S8). Potential gross and histological changes in the major organs of each mouse are shown in Supplementary Fig. S9 and Fig. 8. No apparent inflammatory cell infiltration, HANPs or HANPs-loaded CS/Alg hydrogel remaining were found. The hematological data are shown in Supplementary Table S2. All the measured parameters, including RBC, WBC, PLT, HGB, EOS, NEUT and LYM of the HANPs and HANPs-loaded hydrogel showed no significant difference, compared with the control as well as CS/Alg group. Similarly, the serum biochemical parameters containing ALT, AST, CREA, Ca and Glu showed no significant difference among these groups (Supplementary Table S3).

## Discussion

The rod-shaped HANPs were previously demonstrated to have good application potential for CMM [27]. However, it is difficult to apply HANPs clinically. On the other hand, hydrogels are extremely hydrophilic 3D networks of hydrophilic polymers similar to the extracellular matrix (ECM) [28]. Nevertheless, the poor mechanical strength and defects in the strong burst release of hydrogels limit their application [31, 32]. Therefore, the HANPs hybridized with CS and Alg were used to generate a novel multifunctional scaffold.

As previously described, the tumor inhibition ability of HANPs is extremely correlated to their structural properties, including morphology, size and crystallinity [12]. Thus, it is worth exploring whether characteristics of HANPs before and after encapsulation within hydrogel differ. Recently, our research group has confirmed that HANPs were simply physically encapsulated within hydrogel and there were almost no changes in the particles’ physicochemical and biological properties after release from the hydrogel [34]. Zhang *et al*. also reported that the rod-like HANPs coated porous titanium scaffold showed a prominent effect on suppressing the tumor growth by a considerable amount of released HANPs [23]. In this study, we confirmed the anti-melanoma effect of HANPs-loaded hydrogel *in vivo* animal experiments. First, whether the polymers, including CS/Alg, have anti-tumor ability alone was excluded. Our observation indicated that the CS/Alg hydrogel could not inhibit tumors, with results similar to those in the PBS control group. Then we observed a significant decrease in tumor volume after treatment with the CS/Alg/HANPs hydrogel, similar to the results of HANPs treatment alone (Fig. 6). Interestingly, the onset of the anti-cancer effect of the HANPs-loaded hydrogel was slower than that of HANPs (Fig. 5). Based on the results of our research group mentioned above [23, 34], we speculated that the composite hydrogel exhibited a sustained release behavior. Although the HANPs-loaded hydrogel had no prominent advantage compared with raw HANPs in our experiment, the composite hydrogel demonstrated a significant ability to inhibit growth and activate apoptosis of melanoma. More amounts of animals and prolonged observation times might be required to further clarify the priority of the composite hydrogel. To the best of our knowledge, no previous studies have focused on applying HANPs-loaded hydrogels in CMM without the inclusion of therapeutic molecules.

Apoptosis is a process of programmed cell death. It is well known that the Bcl-2 family controls the intrinsic apoptotic pathway and that the extrinsic pathway is associated with TNF family death factors [35]. Our data *in vitro* indicated that apoptosis induction was accompanied by increased expression of Bax (a proapoptotic protein), Bad (a proapoptotic effector protein) and Cyt C (a protein released from mitochondria into the cytosol) and decreased expression of Bcl-2 (an antiapoptotic protein), suggesting that HANPs induce apoptosis by triggering the mitochondrial apoptotic pathway. DNA damage can induce p53 protein increases, ultimately inducing cell apoptosis to some extent. Increased expression of p53 was also detected in our study (Fig. 2). Histological analysis revealed that the anti-melanoma capability might also be attributed to the mitochondrial apoptotic pathway *in vivo* (Fig. 7). Consistent with our findings, Liu *et al.* found that HANPs promoted apoptosis of the human hepatoma cell line BEL-7402 *invitro* [36]. Zhao *et al.* discovered that in addition to apoptosis, mitochondrial swelling and an increasing number of lysosomes were observed in breast cancer cells (4T1) treated with HANPs [15]. The TEM observations provided compelling evidence that HANPs could enter both tumor cells and normal cells, but the differences may be related to swelling mitochondrial (Fig. 3). Intracellular redox homeostasis is regulated by oxidative and antioxidant substances, mainly in the mitochondria [37]. We found that HANPs exposure caused an obvious increase in the MDA level and a reduction in GSH, CAT and SOD levels. GSH and SOD are the essential antioxidants involved in maintaining redox balance (Supplementary Fig. S4). SOD converts O^2-^ generated via enzymatic reactions to hydrogen peroxide, further aggravating oxidative stress, which would cause MDA, an index of lipid peroxidation [38]. In particular, oxidative stress plays a crucial role in apoptosis [39] and impairs mitochondrial membrane integrity, leading to Cyt C release, caspase activation and apoptosis.

The proteomics technique is a precise method to reveal the DEPs associated with biological processes or diseases and is widely used to investigate complex underlying mechanisms [40, 41]. The presence of mutated β-catenin is associated with aggressive tumor growth and regulates the expression of various target genes that mediate proliferation, apoptosis and migration [42]. DKK1 is an inhibitor of the Wnt/β-catenin pathway. Mikheev *et al.* reported that the overexpression of DKK1 induced apoptosis, thus preventing tumor growth in nude mice [43]. Wnt5a increases cell motility and elevates the levels of free Ca^2+^ in a polarized manner [44]. Furthermore, it antagonizes the Wnt/β-catenin pathway [45]. The anti-tumor effect of IGFBP3 regulates mitogenic and antiapoptotic effects. Its anti-tumor effect is due to inhibition of the Wnt pathway; thus, IGFBP3 treatment is an attractive potential therapeutic approach for melanoma [46]. To date, few reports on IGFBP5 in melanoma have been published. Wang *et al*. confirmed that IGFBP5 might be a tumor suppressor in melanoma [47]. THBS1 has been previously described as an inhibitor of angiogenesis and tumorigenesis [48, 49]. APOA1 potently suppresses tumor growth through innate and adaptive immune processes [50]. Daryoush *et al*. revealed that APOA1 might play a potent immunomodulatory role in the tumor microenvironment, altering tumor-associated macrophages [51]. In accordance with the TMT data, HANPs had significantly elevated Wnt5a, DKK1, IGFBP3, THBS1 and APOA1 protein expression by WB (Fig. 4). Therefore, we speculated that once HANPs were taken up by melanoma cells, abnormal mitochondria and corresponding redox imbalance may occur, further regulating the Wnt, proteoglycans in cancer, p53 and oxidative phosphorylation signaling pathways by mediating Wnt5a, DKK1, IGFBP3, THBS1 and ApoA1 protein expression and ultimately promoting the mitochondrial apoptotic pathway.

The biosafety of nano-materials is of considerable concern [52]. None of the experimental animals died, and no abnormal changes in general condition, such as animal activity or breathing, were observed in this study. No significant differences in hematological parameters (Supplementary Table S2), biochemical analysis (Supplementary Table S3), inflammation infiltration levels or external–internal injury levels were found in the major organs (Fig. 8; Supplementary Fig. S9). Our results indicated that the HANPs-containing hydrogel aligned with the surrounding natural healthy tissue and was biocompatible and safe, unlike traditional chemotherapeutic drugs that damage normal tissue. Based on our work, we speculate that the HANPs-loaded hydrogel possesses an anti-melanoma capability and a possible extended-release feature, indicating the substantial potential for its use as a biomedical material and clinical translation in the near future. Despite these findings, much work, including a material genome initiative based on big data analyses and comparisons with other drugs, is required to illustrate the underlying mechanisms better and provide a stronger foundation for future clinical trials evaluating HANPs-containing hydrogels for the post-surgical treatment of CMM.

## Conclusion

The CS/Alg/HANPs hydrogel was applied in this study to satisfy the actual requirements of CMM treatment. This composite hydrogel had a significant anti-melanoma effect and no prominent toxic effects. HANPs induced intrinsic apoptosis *in vitro* and *in vivo*, as did the HANPs-loaded hydrogel. Furthermore, the anti-melanoma effect might arise from multiple pathways and several target proteins together. Abnormal mitochondria and redox imbalance may occur after HANPs enter melanoma cells, further regulating the Wnt signaling, proteoglycans in cancer, p53 signaling pathways and oxidative phosphorylation via increased Wnt5a, DKK1, IGFBP3, THBS1 and ApoA1 protein expression, ultimately inducing apoptosis. Our findings demonstrate that this HANPs-loaded hydrogel has substantial application potential and provides a stronger foundation for future clinical trials.

## Supplementary data

Supplementary data are available at *REGBIO* online.

## Funding

This work was financially supported by National Key Research and Development Program of China (2017YFB0702600 and 2017YFB0702604) and Sichuan Science and Technology Program (2020YFS0038).

*Conflicts of interest statement.* None declared.

## Supplementary Material

rbac050_Supplementary_Data
