# Supplementary material for: Anti-melanoma effect and action mechanism of a novel chitosan-based composite hydrogel containing hydroxyapatite nanoparticles
Source: Regen Biomater. 2022 Jul 29;9:rbac050. doi: 10.1093/rb/rbac050 (PMC9362996; doi:10.1093/rb/rbac050)
Supplement: rbac050_Supplementary_Data [file rbac050_supplementary_data.zip › Supplementary data/Electronic Supplementary Materials.docx]

**Electronic Supplementary Materials**

Fig. s1. Morphology was observed in SK-MEL-28 and A375 cells with treated HANPs at various concentrations by Hoechst 33258 staining. The arrows indicate nuclear shrinkage, condensation and fragmentation. Original magnification is × 200.

Fig. s2. In vitro cell apoptosis cocultured with HANPs suspensions at various concentrations in HaCaT cells. Hoechst 33258 (a), Annexin V-FITC/PI apoptosis detection results (b) and Western blotting analysis were performed (c).

Fig. s3. TEM micrographs of treatment with HANPs in HaCaT cell.

Fig. s4. Effects of HANPs on oxidative stress in SK-MEL-28, A375 and HaCaT cells.

Fig. s5. Volcano map of DEPs (HANPs versus control).

Fig. s6. H&E staining and IF staining of excised tumor tissues. (a)The histological analysis was presented. Green arrows indicating presence of nanohydroxyapatite, red arrows representing presence of patchy edema and less tumor cells, yellow arrows demonstrating presence of eosin-like staining speculated as hydrogel matrix. Scale bars, 20 µm. (b) The TUNEL assay were performed. Scale bars, 20 µm.

Fig. s7. Quantification of immunohistochemical staining of Bax, Bcl-2, cyt-c and p53 expression of tumor tissues.

Fig. s8. Animal body weight changes during the experiment

Fig. s9. Gross observation of major organs including heart, liver, spleen, kidney and lung of each mouse at the endpoint.

Table s1. The top 40 tumor-related proteins between HANPs treatment (240 μg/mL) and control group (0 μg/mL) by TMT.

Table s2. The hematology parameters in SK-MEL-28 and A375 melanoma models.

Table s3. The serum biochemical parameters in SK-MEL-28 and A375 melanoma models.
