# Supplementary figures and images for: Anti-melanoma effect and action mechanism of a novel chitosan-based composite hydrogel containing hydroxyapatite nanoparticles
Source: Regen Biomater. 2022 Jul 29;9:rbac050. doi: 10.1093/rb/rbac050 (PMC9362996; doi:10.1093/rb/rbac050)

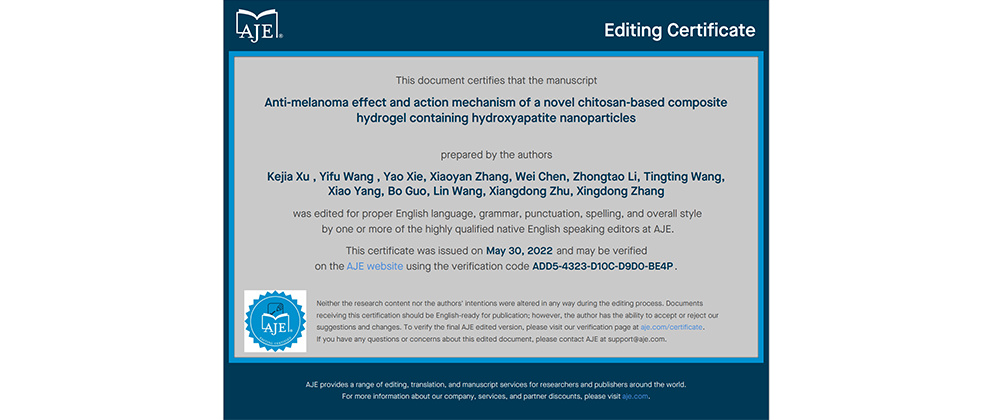

Supplement: rbac050_Supplementary_Data [file rbac050_supplementary_data.zip › Supplementary data/Edit certificate.tif]

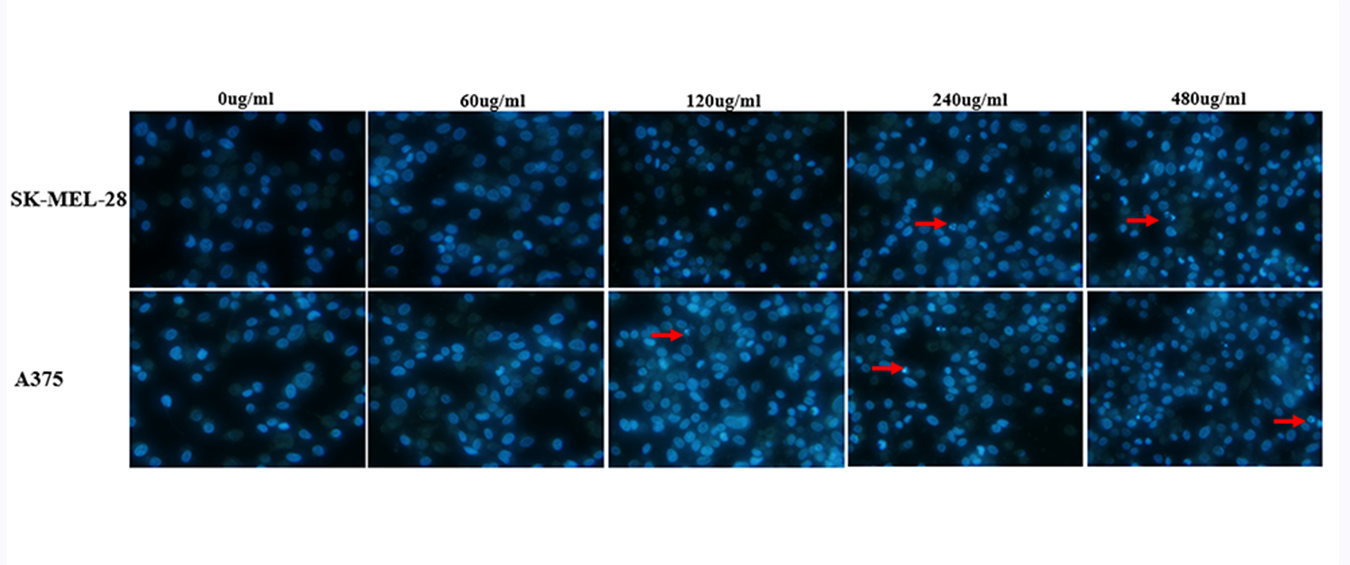

Supplement: rbac050_Supplementary_Data [file rbac050_supplementary_data.zip › Supplementary data/Fig.s1.tif]

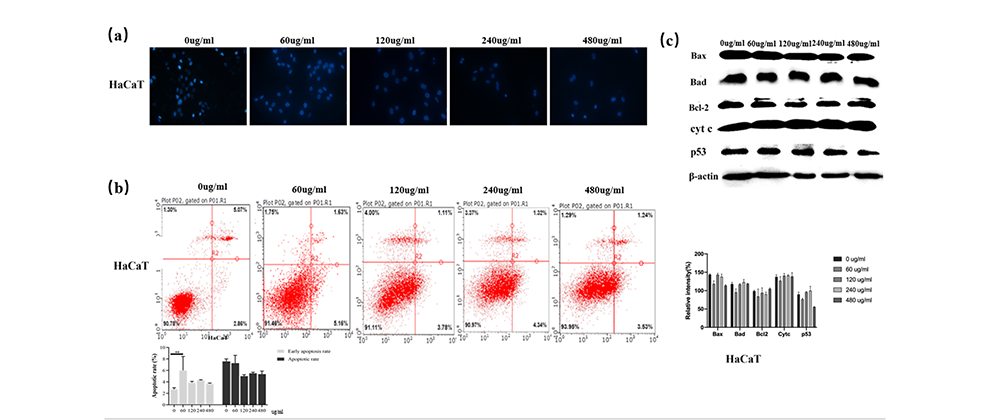

Supplement: rbac050_Supplementary_Data [file rbac050_supplementary_data.zip › Supplementary data/Fig.s2.tif]

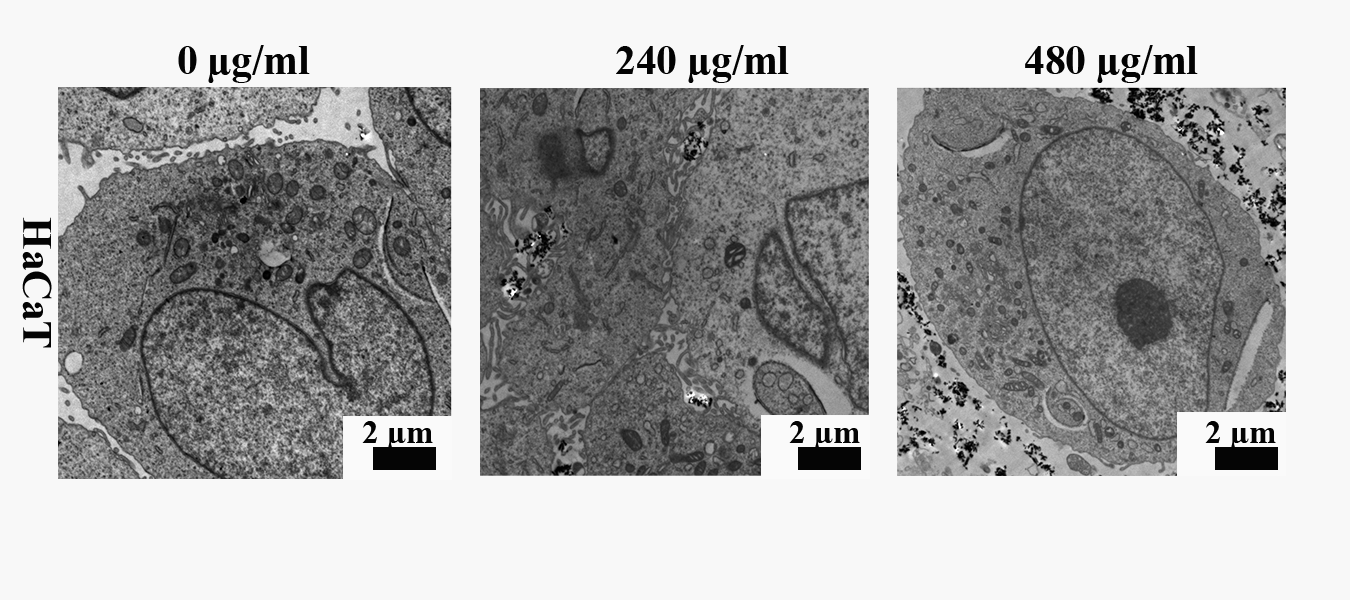

Supplement: rbac050_Supplementary_Data [file rbac050_supplementary_data.zip › Supplementary data/Fig.s3.tif]

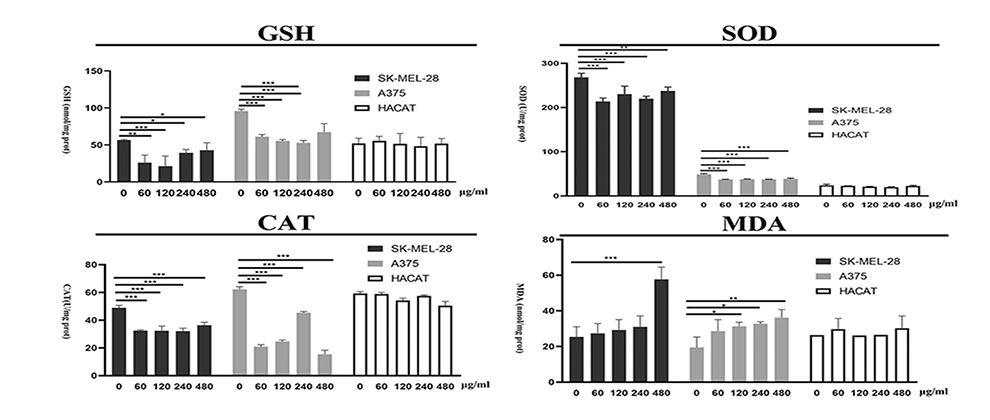

Supplement: rbac050_Supplementary_Data [file rbac050_supplementary_data.zip › Supplementary data/Fig.s4.tif]

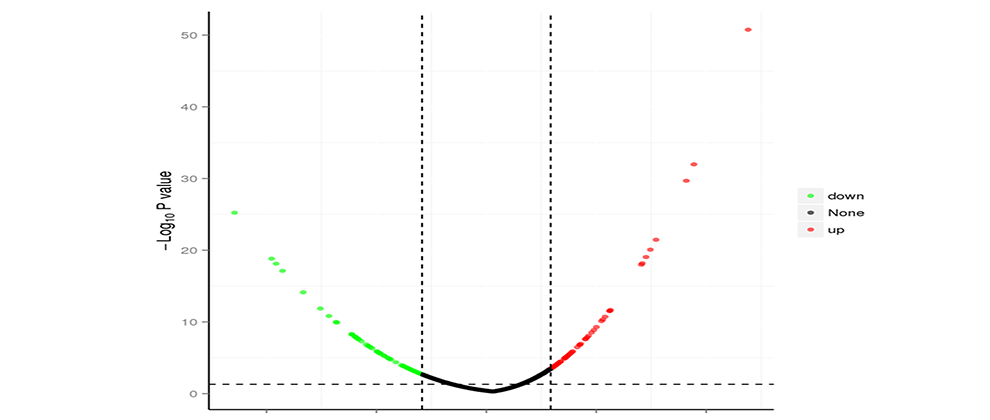

Supplement: rbac050_Supplementary_Data [file rbac050_supplementary_data.zip › Supplementary data/Fig.s5.tif]

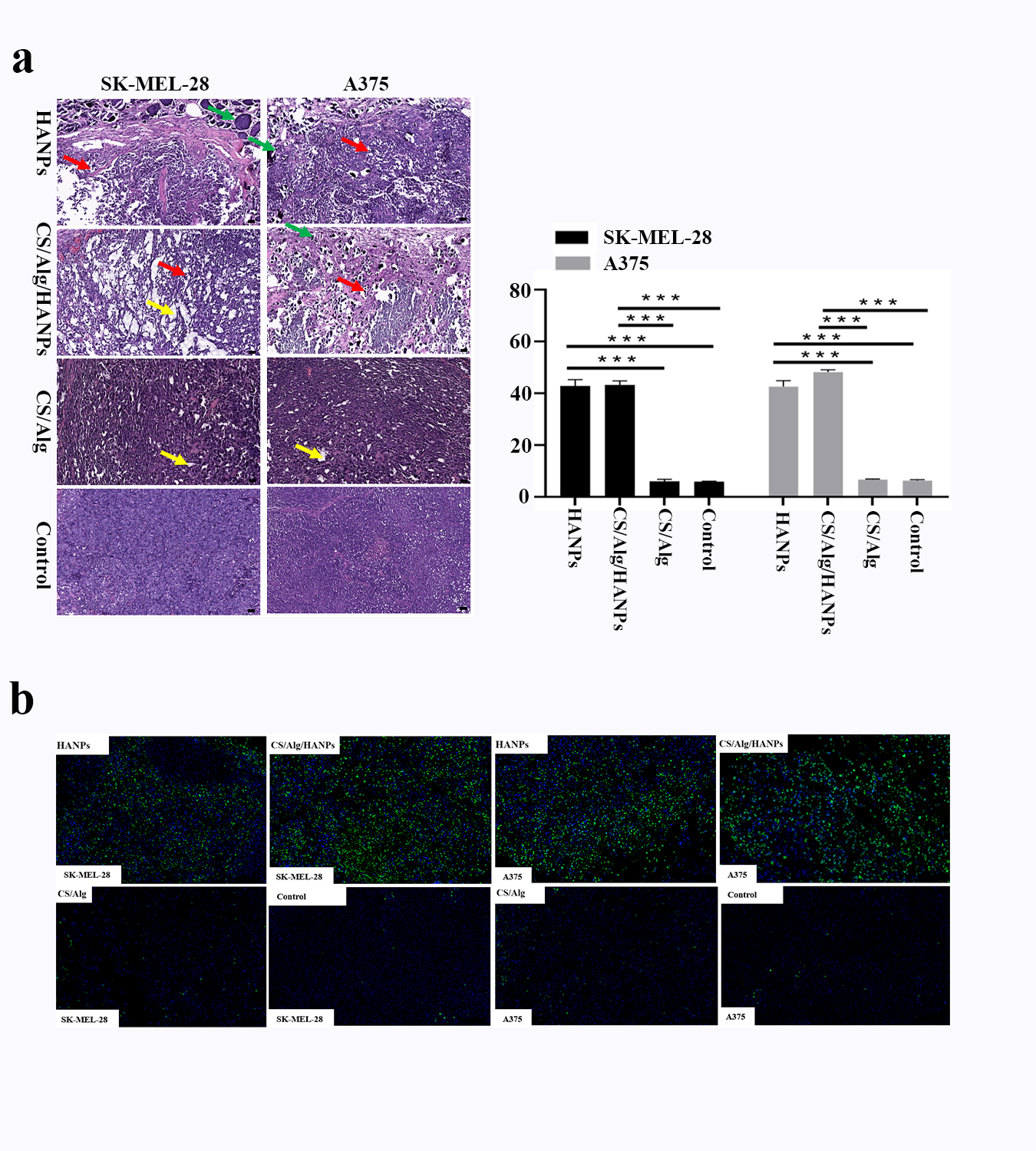

Supplement: rbac050_Supplementary_Data [file rbac050_supplementary_data.zip › Supplementary data/Fig.s6.tif]

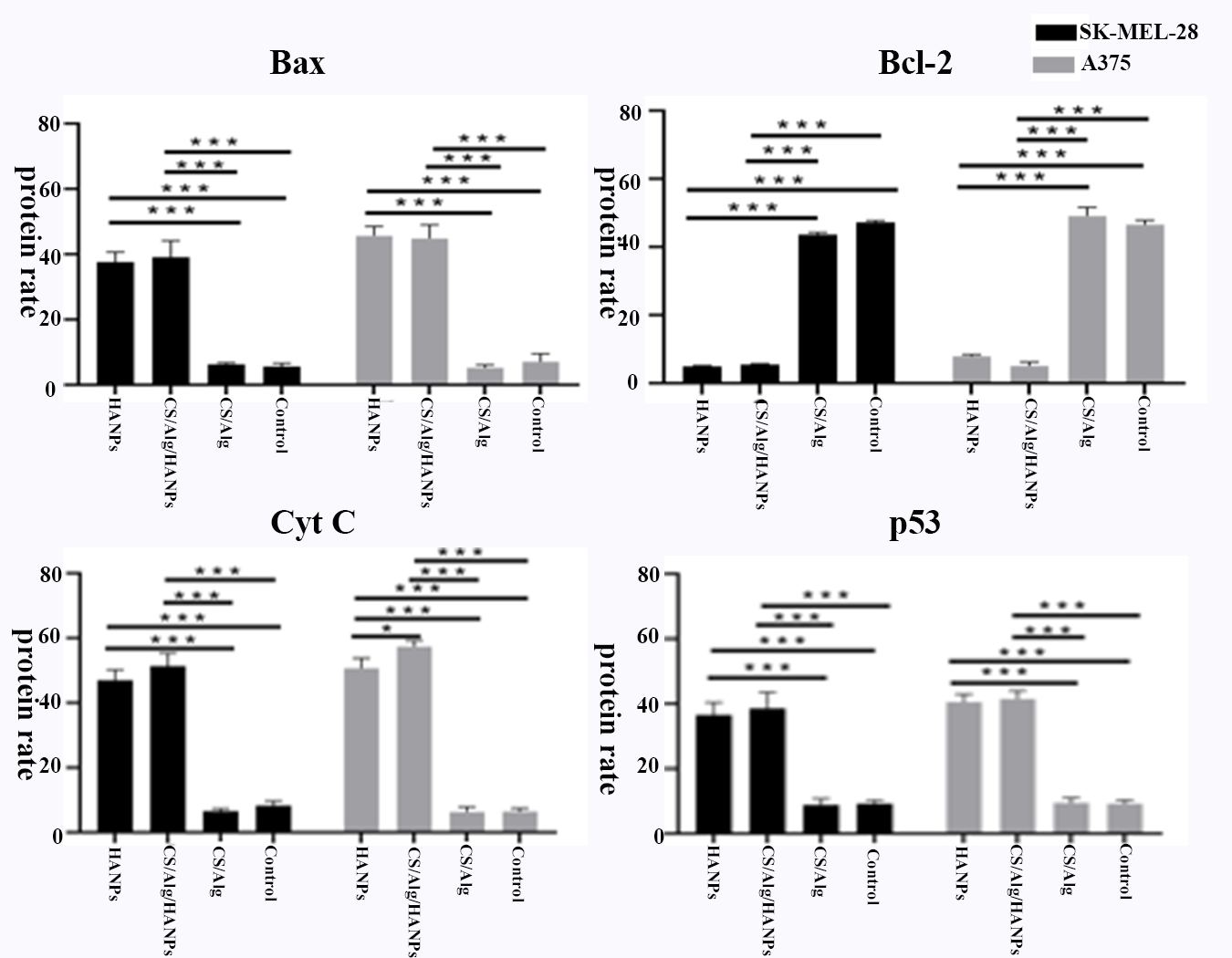

Supplement: rbac050_Supplementary_Data [file rbac050_supplementary_data.zip › Supplementary data/Fig.s7.tif]

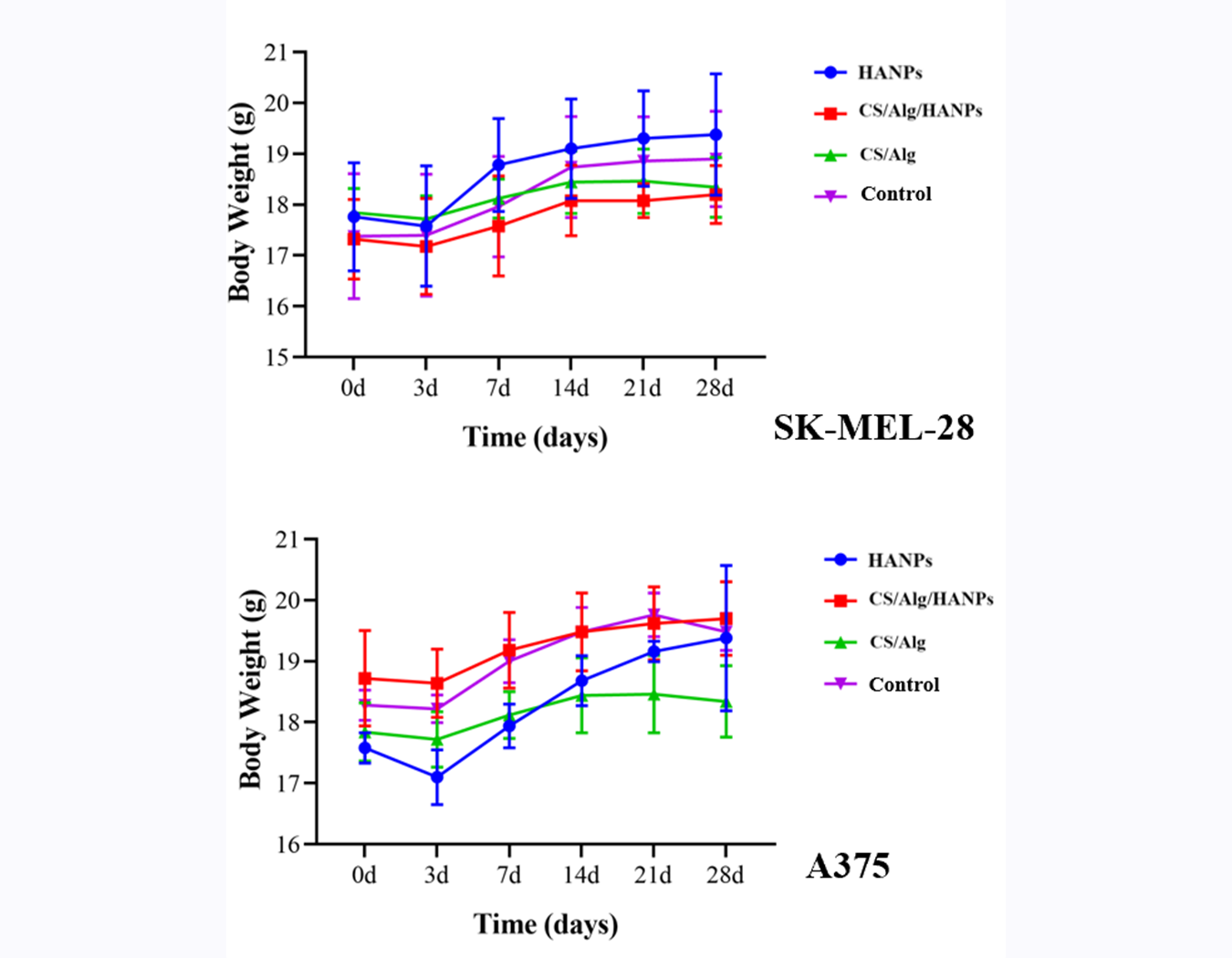

Supplement: rbac050_Supplementary_Data [file rbac050_supplementary_data.zip › Supplementary data/Fig.s8.tif]

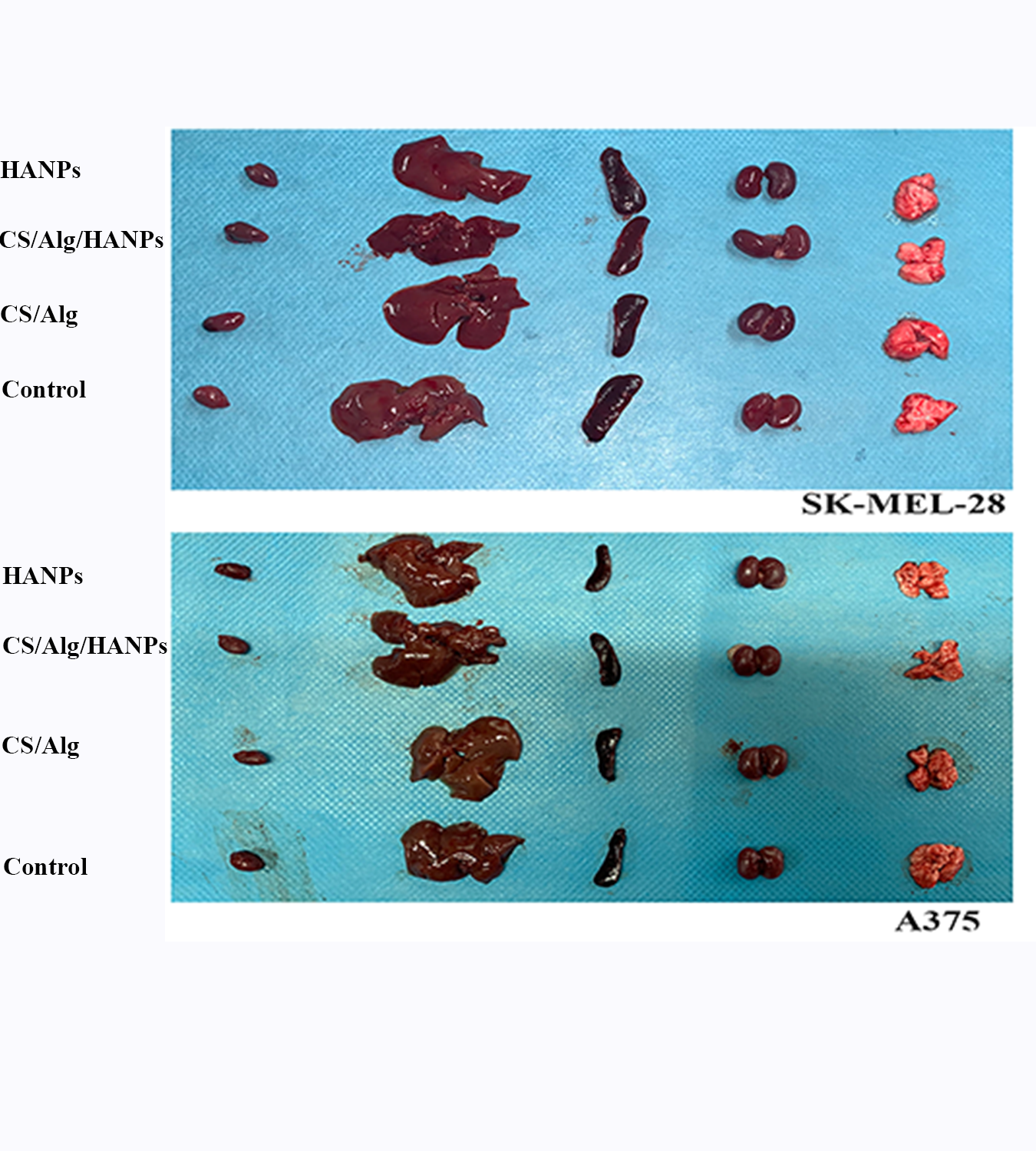

Supplement: rbac050_Supplementary_Data [file rbac050_supplementary_data.zip › Supplementary data/Fig.s9.tif]

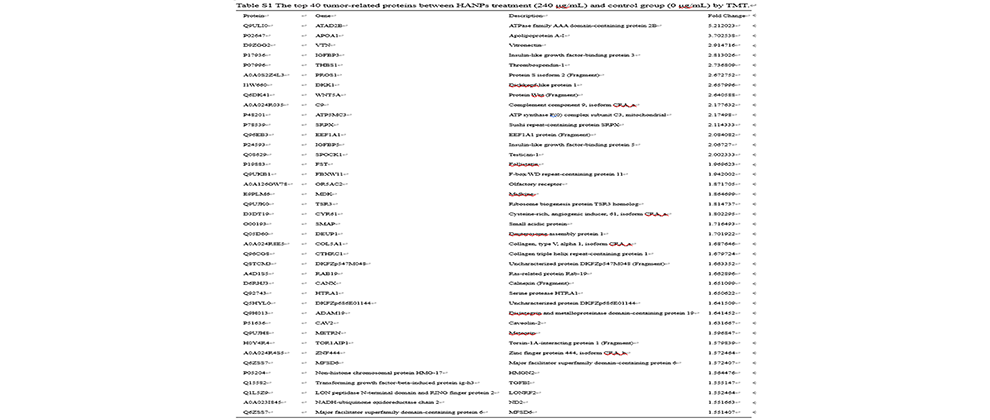

Supplement: rbac050_Supplementary_Data [file rbac050_supplementary_data.zip › Supplementary data/Table.s1.tif]

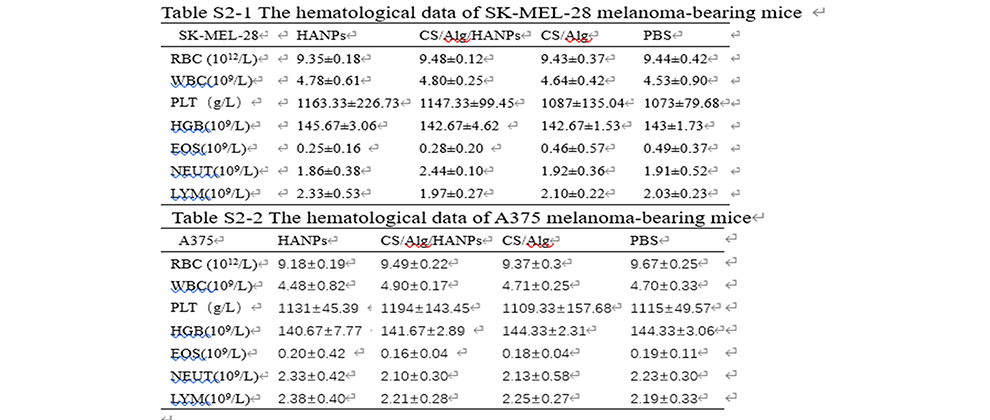

Supplement: rbac050_Supplementary_Data [file rbac050_supplementary_data.zip › Supplementary data/Table.s2.tif]

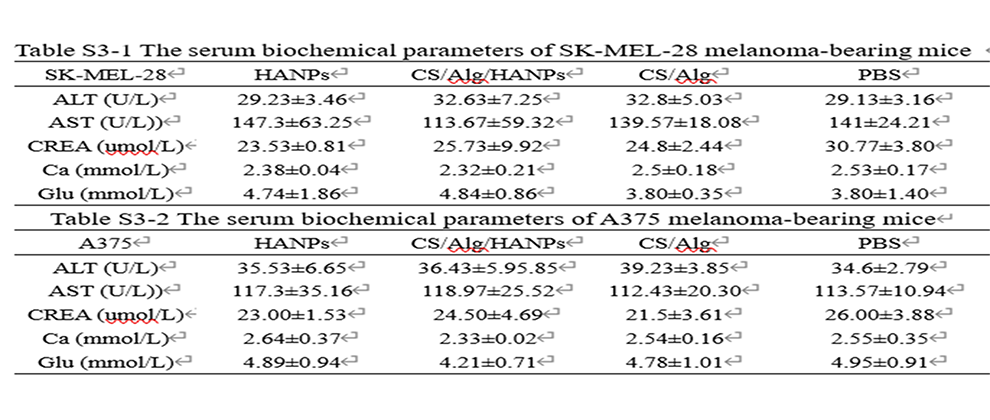

Supplement: rbac050_Supplementary_Data [file rbac050_supplementary_data.zip › Supplementary data/Table.s3.tif]
